# Kv7.4 Channel Contribute to Projection-Specific Auto-Inhibition of Dopamine Neurons in the Ventral Tegmental Area

**DOI:** 10.3389/fncel.2019.00557

**Published:** 2019-12-18

**Authors:** Min Su, Li Li, Jing Wang, Hui Sun, Ludi Zhang, Chen Zhao, Ying Xie, Nikita Gamper, Xiaona Du, Hailin Zhang

**Affiliations:** ^1^Department of Pharmacology, Hebei Medical University, Shijiazhuang, China; ^2^The Key Laboratory of Neural and Vascular Biology, Ministry of Education, Shijiazhuang, China; ^3^The Key Laboratory of New Drug Pharmacology and Toxicology, Shijiazhuang, China; ^4^Department of Pharmacochemistry, Hebei University of Chinese Medicine, Shijiazhuang, China; ^5^Center for the Experimental Animal, Hebei Medical University, Shijiazhuang, China; ^6^Faculty of Biological Sciences, University of Leeds, Leeds, United Kingdom

**Keywords:** Kv7/M channels, Kir3/GIRK channel, ventral tegmental area, dopamine neuron, D2 receptor, circuit, neuronal excitability

## Abstract

Dopaminergic neurons in the ventral tegmental area (VTA) encode behavioral patterns important in reward and drug addiction as well as in emotional disorders. These functions of dopamine neurons are directly related to the release of dopamine in the targeted regions of the brain which are, thus, controlled by the excitability of dopamine neurons. One mechanism for modulation of dopamine neuronal excitability is mediated by the auto dopamine type 2 (D2) receptors, through activation of a Kir3/GIRK K^+^ channel which inhibits the firing of dopamine neurons. In this study, we provide evidence that Kv7.4, in addition to Kir3.2 channels, contributes to dopamine (DA)-mediated auto-inhibition of DA activity projecting to NAc and to basolateral amygdale (BLA). Furthermore, we demonstrate that D2 receptors enhance Kv7.4 currents through G_i/o_ protein and redox-dependent cellular pathway. Finally, we show this D2 mediated auto-inhibition is blunted in a social defeat mice model of depression, a phenomenon that may contribute to the altered excitability of VTA DA neurons in depressed animals. These results provide a new perspective for understanding the molecular mechanism of the excitability of VTA DA neurons and for potential new strategies against mental disorders involving altered excitability of DA neurons, such as major depression and drug addictions.

## Introduction

Recent studies suggested that ventral tegmental area (VTA) dopamine (DA) neurons in the mesocorticolimbic system are integrated into different circuits, including different subregions of the nucleus accumbens (NAc), medial prefrontal cortex (mPFC), and the amygdala (Lammel et al., 2008, 2011; Chaudhury et al., 2013).

VTA DA neurons is a functionally heterogeneous population, which is reflected in distinct firing (Krashia et al., 2016). Firing pattern of VTA DA neurons is directly related to the release of DA and thus is the direct determinant of dopaminergic function (Beckstead et al., 2007; Courtney et al., 2012; Ford, 2014). These DA neuron firing patterns are crucial for the behavioral outcomes of VTA activity. However, ionic mechanisms for modulation of VTA DA neurons excitability and firing patterns are not fully understood.

Somatodendritic DA release in the VTA decreases the excitability of dopamine neurons through activating D2-autoreceptors on DA neurons (Beart et al., 1979). This auto modulation involves K^+^ channels of Kir family (G-protein gated inwardly rectifying K^+^ channels, GIRK), which are linked to D2 receptors by G_i/o_ type of G protein, leading to hyperpolarization of the cells (Beaulieu and Gainetdinov, 2011). Recent work suggests that VTA-mPFC DA neurons lack D2 receptor-mediated auto-inhibition (Lammel et al., 2008), while the NAc- and basolateral amygdale (BLA)-projecting DA neurons express somatodendritic D2 receptors and Kir3 channels robustly (Ford et al., 2006).

Another K^+^ channel family that appears to be an important regulator of VTA DA firing/activity is the Kv7/KCNQ channels (Jentsch, 2000; Li et al., 2017). Four members of Kv7/KCNQ channels (Kv7.2–7.5) are expressed in the CNS (Jentsch, 2000). Stimulation of Kv7 channels leads to inhibition of neuronal activity (Hansen et al., 2008; Drion et al., 2010). It has been shown dopamine D2 receptors and Kv7/KCNQ channels are co-localized in postsynaptic regions of several mammalian brain regions (Ljungstrom et al., 2003). Stimulation of co-expressed D2 receptors increases the Kv7/KCNQ currents in a heterologous expression system (Ljungstrom et al., 2003), indicating a potential mechanism of Kv7 activation in D2 receptor-mediated auto-inhibition of DA neurons which might be employed in addition to a well-known D_2_R-Kir3/GIRK mechanism, especially in neurons with low levels of Kir3/GIRK channel expression.

Earlier findings by us (Li et al., 2017) and others (Hansen et al., 2006) demonstrated that the Kv7.4 subunit of Kv7 is selectively expressed in the midbrain, especially in the VTA (Li et al., 2017). The aim of this study was 3-fold: (i) to determine the projection-specific expression pattern of Kv7.4 in VTA DA neurons; (ii) to identify Kv7.4 as a target of D2 receptor-mediated auto-inhibition of VTA DA neurons; and (iii) to probe the involvement of D2-Kv7.4 pathway mechanism in a social defeat mouse model of depression.

## Materials and Methods

### Mouse Lines and Retrograde Labeling

C57BL/6 mice (Vital River, China, male, 8–10 weeks old) were used for the experiments. Kv7.4^−/−^ mice were provided by *Prof Thomas Jentsch* (FMP, MDC, Berlin, Germany; Kharkovets et al., 2006). Kir3.2 (Gene ID: 16522) knock-out mice were produced by Biocytogen Company (Beijing, China). All experiments were conducted in accordance with the guidelines of the Animal Care and Use Committee of Hebei Medical University and approved by the Animal Ethics Committee of Hebei Medical University. Neuroanatomical nomenclature as described in the Franklin and Paxinos mouse brain atlas (Paxinos and Franklin, 2001). Red fluorescent retrobeads (100 nl for single injection; Lumafluor Inc., Naples, FL, USA) were injected into NAc core (AP +1.50, LM 0.84, DV −4.0; 100 nl beads) and BLA (AP −1.46, LM 2.85, DV −4.3; 100 nl beads); mPFC was injected at four separate sites (AP +2.05 and 2.15, LM 0.27, DV −2.1 + 1.7; 200 nl beads). Retrobeads were delivered at a rate of 100 nl/min and left for at least 5 min after injection. For sufficient labeling, survival periods for retrograde tracer transport depended on respective injection areas: NAc core, 14 days; mPFC, 21 days; BLA 14 days.

### Electrophysiological Recordings

The details of coronal VTA brain slice preparation were the same as our previously published work (Li et al., 2017). Recordings in the slices were performed in whole-cell current-clamp and voltage-clamp configurations on an Axopatch 1D amplifier coupled with a Digidata 1440A AD converter (Molecular Devices, San Jose, CA, USA). For Kv7/M current recording, neurons were held at −25 mV, and then 1 s square pulses to −50 mV were used repeatedly with a 20 s interval. Kv7/M current was measured as the instantaneous deactivating tail current at the beginning of a voltage step to −50 mV (Koyama and Appel, 2006). Neurons exhibiting no or irregular spontaneous activities were not evaluated. Firing rates were analyzed by counting the number of action potentials within 1-min time windows. Two brain slices were moved to a 5 ml superfusion system added PTX for 6–9 h at room temperature (22–25°C) until use. The control experiment did not add PTX in artificial cerebrospinal fluid (ACSF).

### Immunohistochemistry

Processes of immunohistochemistry were performed as previously described (Li et al., 2017), with some modifications. The DAPI-stained coronal midbrain sections (200 μm-thick) from mice that were injected with retrobeads were reconstructed with CaseViewer (Pannoramic MIDI, 3DHISTECH, Hungary). Primary antibodies: mouse anti-TH (tyrosine hydrolyze; 1:400, Merck Millipore, Darmstadt, Germany), rabbit anti-Kv7.4 (1:100, AlomoneLabs, Jerusalem, Israel), goat anti-Kir3.2 (1:400, Santa Cruz Biotechnology, Santa Cruz, CA, USA). Following secondary antibodies were used: FITC-conjugated AffiniPure donkey anti-goat IgG (Jackson ImmunoResearch, West Grove, PA, USA; 1:400), Cy3-conjugated AffiniPure donkey anti-rabbit IgG (Jackson ImmunoResearch, West Grove, PA, USA; 1:400) and Cy5-conjugated AffiniPure donkey anti-mouse IgG (Jackson ImmunoResearch, West Grove, PA, USA; 1:400). Images were obtained on a Leica TCS SP5 confocal laser microscope (Leica, Germany).

### Chronic Social Defeat Stress

Complete experimental methods for chronic social defeat model were described in our previously published work (Li et al., 2017).

### Single-Cell PCR

Methods for single-cell polymerase chain reaction (PCR) were described in our previously published work (Li et al., 2017). Then two rounds of conventional PCR with pairs of gene-specific primer pairs into each PCR tube.

The “outer” primers (from 5′ to 3′) were as follows:

**Table:** 

GAPDH	AAATGGTGAAGGTCGGTGTGA ACG (sense)	AGTGATGGCATGGACTGT GGT CAT (antisense)
TH	GCCGTCTCAGAGCAGGATAC	GGGTAGCATAGAGGCCCTTC
DAT	CTGCCCTGTCCTGAAAGGTGT	GCCCAGTGATCACAGACTCC
D2	AGCATCGACAGGTACACAGC	CCATTCTCCGCCTGTTCACT
Kv7.4	CCCGGGTGGACCAAATTGT	AGCCCTTCAGTCCATGTTGG
Kir3.2	TGGACCAGGATGTGGAAAGC	AAACCCGTTGAGGTTGGTGA

The “inner” primers (from 5′ to 3′) were as follows:

**Table:** 

GAPDH	GCAAATTCAACGGCACAG TCAAGG	TCTCGTGGTTCACACCCA TCACAA
TH	AGGAGAGGGATGGAAATGCT	ACCAGGGAACCTTGTCCTCT
DAT	ATTTTGAGCGTGGTGTGCTG	TGCCTCACAGAGACGGTAGA
D2	CCATTGTCTGGGTCCTGTCC	GTGGGTACAGTTGCCCTTGA
Kv7.4	ATGGGGCGCGTAGTCAAGGT	GGGCTGTGGTAGTCCGAGGTG
Kir3.2	AGCCGAGACAGGACCAAAAG	ATGTACGCAATCAGCCACCA

### Statistical Analysis

We used *t*-tests to examine the effects of the drugs on the measured parameters among individual neurons. Repeated measures one-way ANOVA with Bonferonni correction was used for multiple groups in the significance of changes in discharge rate and currents. Wilcoxon signed-rank test was used to analyze the normalized firing frequency based on the data. *p*-values ≤ 0.05 were accepted as significant.

## Results

### Kv7.4 Contributes to DA-Induced Inhibition on the Firing of NAc-Projecting VTA DA Neurons

Using retrograde beads and TH antibody for labeling DA neurons, we found that 69%, 60% and 75% TH positive neurons project to NAc (VTA-NAc), mPFC (VTA-mPFC) and BLA (VTA-BLA), respectively. DA (20 μM) produced similar inhibitory effects on the firing of NAc- and BLA-projecting VTA DA neurons. On the other hand, DA had little effects on the firing of DA neurons projecting to mPFC (data are not shown), likely resulting from the absence of D2-receptor (Lammel et al., 2008).

**Figure 1:**
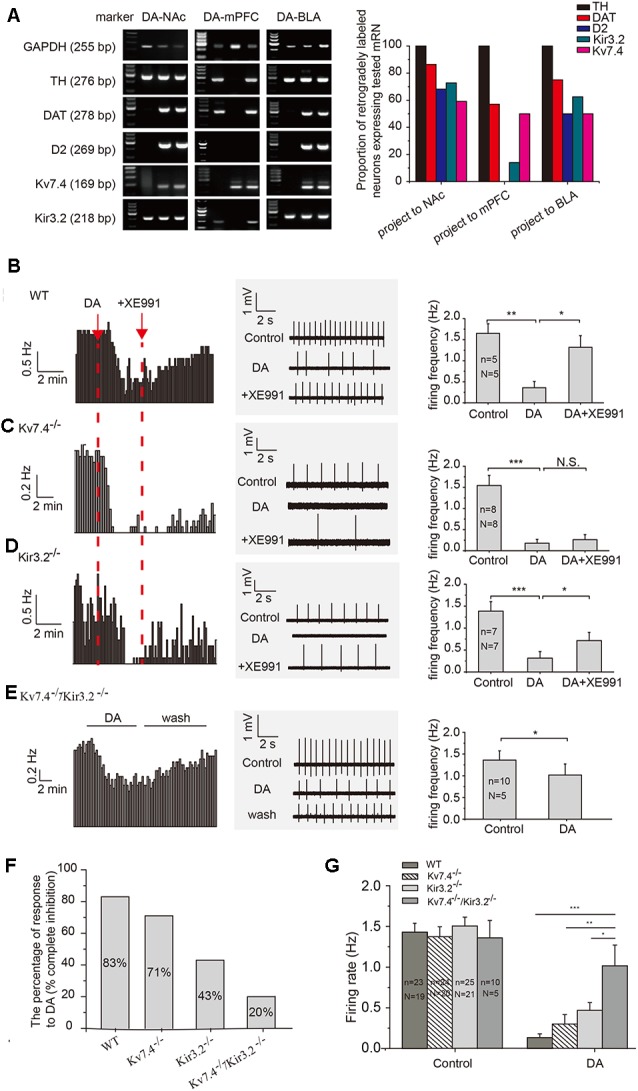
Kv7.4 contribute to the dopamine (DA)-induced inhibition of NAc-projecting DA neuron firing. **(A)** Single-cell polymerase chain reaction (PCR) analysis in retrogradely labeled ventral tegmental area (VTA) DA neurons from different projections. **(B–D)** VTA DA neuron firing recorded with loose cell-attached patch recordings. XE991 (3 μM), a Kv7 blocker, reversed the DA-induced inhibition of neuron firing in WT **(B)** and Kir3.2^−/−^ **(D)** mice, but not in Kv7.4^−/−^ mice **(C)**. **(E)** The effect of DA on spontaneous firing in DA neurons from Kv7.4^−/−^/Kir3.2^−/−^ mice. **(F)** The percentage of VTA-NAc DA neurons with firing rate being inhibited to less than 20% (complete inhibition) in WT, Kv7.4^−/−^, Kir3.2^−/−^ and Kv7.4^−/−^/Kir3.2^−/−^ mice. **(G)** Average DA inhibition on firing rate in all recorded VTA-NAc DA neurons from WT, Kv7.4^−/−^, Kir3.2^−/−^ and Kv7.4^−/−^/Kir3.2^−/−^ mice. One-way repeated-measures ANOVA with Bonferroni *post hoc* test. ***p < 0.001, **p < 0.01, *p < 0.05; N.S., not significant. One-way repeated-measures ANOVA with Bonferroni *post hoc* test. *N*, number of animals; *n*, number of recordings.

We used a single-cell PCR method to study the expression pattern of the D2 receptor, as well as the Kv7.4 and Kir3.2 channels in VTA DA neurons. Only TH positive DA neurons were evaluated. As has been reported (Lammel et al., 2011), we found majority of the NAc core- and BLA-projecting DA neurons were positive for DAT and D2; in contrast, D2 receptor mRNA was absent in the mPFC-projecting DA neuron (Figure 1A). As for two K^+^ channels, the expression of Kv7.4 mRNA in projection-specific DA neurons is similarly high: DA-NAc (59%), DA-BLA (50%) and DA-mPFC (50%; Figure 1A, right panel, *n* = 22, 16 and 14 cells respectively); the proportions of Kir3.2-positive neurons were as follows: DA-NAc (73%), DA-BLA (63%) and DA-mPFC (14%; Figure 1A), respectively.

Kir3.2 has been long hypothesized as a mechanism contributing to the auto-inhibition of VTA DA neurons (Arora et al., 2010). Thus, our major purpose of this study was to test the involvement of Kv7.4 in auto-inhibition and compare its contribution to that of Kir3.2, in the VTA DA neurons projecting to NAc, because of the prominent role of this projection in the development of depression behavior (Cao et al., 2010; Chaudhury et al., 2013). In wild-type (WT) mice, the DA-induced inhibition of firing was mostly reversed by selective Kv7 blocker XE991 (3 μM, Alomone Labs, Jerusalem, Israel), indicating a prominent involvement of Kv7.4 (Figure 1B, one-way repeated measures ANOVA, *p* = 0.0305, Bonferroni test). In Kv7.4^−/−^ mice, the DA effects were not significantly affected by XE991 (Figure 1C, one-way repeated measures ANOVA, *p* = 1, Bonferroni test), indicating a mechanism other than Kv7.4 was involved (possibly Kir3.2, see below) and supporting the notion that XE991 as a reliable Kv7.4 blocker. In Kir3.2^−/−^ mice, the DA effect was partly reversed by XE911, in general in agreement with the above results (Figure 1D, one-way repeated measures ANOVA, *p* = 0.0118, Bonferroni test).

In general, DA (20 μM) significantly reduced the average firing frequency of VTA DA-NAc neurons in WT mice (Figure 1B), Kv7.4^−/−^ mice (Figure 1C), Kir3.2^−/−^ mice (Figure 1D) and Kv7.4^−/−^/Kir3.2^−/−^ mice (Figure 1E). The effects of DA were also analyzed in a different way: the percentage of neurons whose firing frequency was reduced by DA to less than 20% of the original firing frequency was counted. In this case, 83% (19/23 cells) of neurons in WT mice, 71% (17/24 cells) of neurons from Kv7.4^−/−^ mice, 43% of neurons (10/23 cells) from Kir3.2^−/−^ mice, and 20% (2/10 cells) of neurons from Kv7.4^−/−^/Kir3.2^−/−^ mice were identified (Figure 1F). The percentage of DA inhibition in all DA treated neurons was also summarized (Figure 1G), and DA effect in Kv7.4^−/−^/Kir3.2^−/−^ mice was significantly smaller than that in Kv7.4^−/−^ mice and Kir3.2^−/−^ mice (Figure 1G), indicating a dominant although not exclusive role of Kv7.4 and Kir3.2 in DA-induced inhibition of VTA DA neuron firing.

### DA Activates Kv7.4 and Kir3.2 Currents

**Figure 2:**
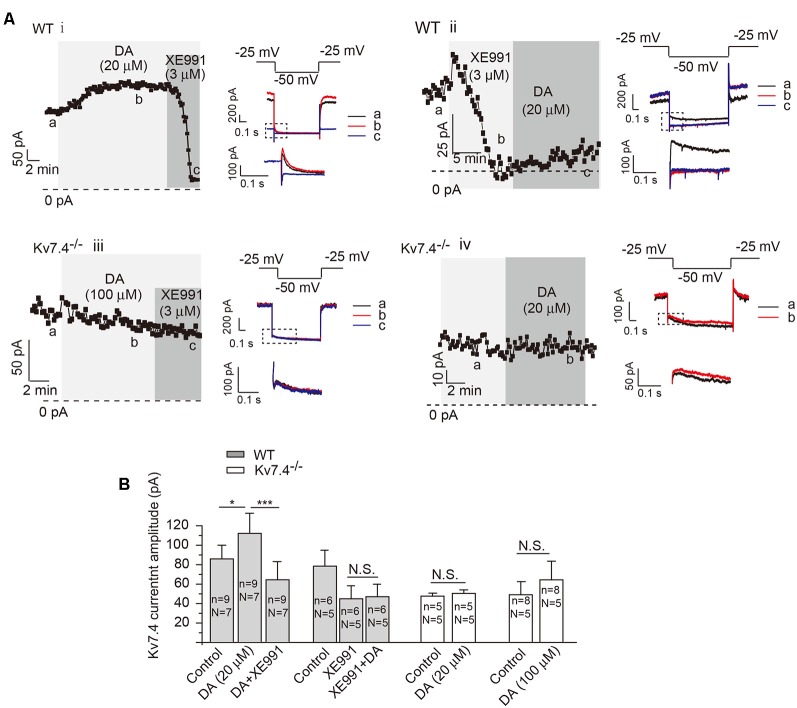
Dopamine activates Kv7/M current in NAc-projecting VTA DA neurons. **(A)** Kv7/M currents recorded from DA neurons of WT **(i,ii)** and Kv7.4^−/−^ **(iii,iv)** mice. Shown are time-courses of M current amplitudes recorded using whole-cell patch-clamp from VTA slices (for details, see “Materials and Methods” section). Summarized data are presented in **(B)**. Error bars represent SEM. ***p < 0.001, *p < 0.05; N.S., not significant. One-way repeated-measures ANOVA with Bonferroni test for WT mice, paired *t*-test for Kv7.4^−/−^ mice. *N* = number of animals; *n*, number of recordings.

To further correlate the DA-induced inhibition with modulation on Kv7.4 channels, we examined the effects of DA on Kv7.4 currents in NAc. In NAc-projecting DA neurons, DA (20 μM) potentiated Kv7/M-like currents, which were blocked by XE911 (3 μM, applied either before or after DA; Figures 2Ai,ii). Kv7 currents were recorded as the characteristic slow deactivating tail current at −50 mV (Koyama and Appel, 2006), which was inhibited by the specific Kv7 channel blocker XE911 at a selective concentration of 3 μM (Figure 2Aii). At the voltage range (−20 ~ −50 mV) and a low K^+^ concentration used to record Kv7/M currents, little Kir3/GIRK currents were activated. Accordingly, the tail deactivating current was inhibited by the specific Kv7 channel blocker XE911 at a selective concentration of 3 μM (Figures 2Ai,ii). This slowly-deactivating XE991-blocked M-like current was completely absent in neurons from Kv7.4^−/−^ mice (Figures 2Aiii,iv,B, paired *t*-test); DA application did not reveal or increase any such current in Kv7.4^−/−^ mice even at a higher concentration (100 μM; Figures 2Aiii,B). These results strongly suggest that DA activates Kv7.4.

Similar experiments were test to observe the role of Kv7.4 in DA-induced inhibition and to study DA activation of Kv7.4 in DA neurons projecting to BLA (Supplementary Figure S1). Similar qualitative conclusions to DA neurons projecting to NAc can be drawn for these BLA-projection DA neurons.

### Testing the Mechanism of DA-Induced Activation of Kv7.4

**Figure 3:**
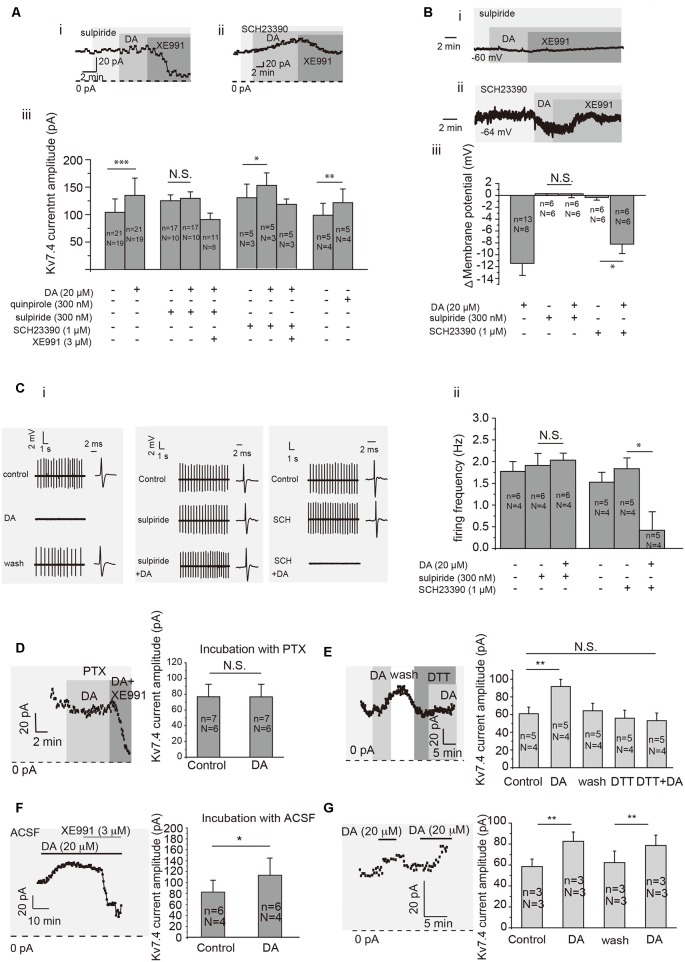
Dopamine activates Kv7/M in VTA DA neurons through dopamine D2 receptor and related cell signaling. **(Ai,ii)**. Example of time-course of Kv7/M currents activated by DA in the presence of D2 receptor inhibitor sulpiride (300 nM) and D1 receptor inhibitor SCH23390 (1 μM). **(Aiii)** Summarized effects of D2 receptor agonist quinpirole (300 nM), sulpiride (300 nM), SCH23390 (1 μM) and Kv7/M channel inhibitor XE991 (3 μM) on DA-induced activation of Kv7/M currents in VTA DA neurons. ***p < 0.001, **p < 0.01, *p < 0.05; N.S., not significant, paired *t*-test. *N* = number of animals; *n* = number of recordings. **(B)** Effect of sulpiride (300 nM) and SCH23390 (1 μM; **i**,**ii**) on the resting membrane potential of VTA DA neurons. Summarized effects of sulpiride and SCH23390 on DA-induced hyperpolarization of VTA DA neurons **(iii)**. *p < 0.05; N.S., not significant, One-way repeated-measures ANOVA with Bonferroni test. **(C)** Example traces from cell-attached recordings of VTA DA neurons activated by DA in the presence of sulpiride and SCH23390 are shown in **(Ci)**. **(Cii)** Summarized effects of DA, sulpiride and SCH23390 on DA-induced inhibition of the spontaneous firing in VTA DA neurons. *p < 0.05; N.S., not significant, paired *t*-test. **(D)** Pre-treating VTA DA neurons with Pertussis toxin (PTX) for 6–9 h abolished the DA-induced activation of Kv7/M channels. N.S, not significant; paired *t*-test. **(E)** Reducing agent dithiothreitol (DTT) blocked the DA-induced activation of Kv7/M channels in VTA DA neurons. ***p < 0.01; N.S. not significant; one-way repeated-measures ANOVA with Bonferroni test. **(F)** Pretreating VTA DA neurons with ACSF for 6–9 h did not affect the DA-induced activation of Kv7/M channels. *p < 0.05; paired *t*-test. **(G)** Repeated application of DA induced a similar degree of Kv7/M activation. **p < 0.01, paired *t*-test. *N* = number of animals; *n* = number of recordings.

We determined that DA modulation of Kv7.4 current in VTA DA neurons was mediated by D2 receptors. First, sulpiride (D2 receptor blocker, Sigma–Aldrich, St. Louis, MO, USA) but not SCH23390 (D1 receptor blocker, Sigma–Aldrich, St. Louis, MO, USA) blocked the DA-induced activation of Kv7.4 currents (Figures 3Ai,iii for sulpiride, *p* = 0.1202, paired *t*-test; Figures 3Aii,iii for SCH23390, *p* = 0.0149, paired *t*-test), DA-induced membrane hyperpolarization (Figures 3Bi–iii) and firing inhibition (Figures 3Ci,ii, *p* = 0.9764, paired *t*-test for sulpiride; *p* = 0.0116, paired *t*-test for SCH23390) of VTA DA neurons. Further, quinpirole (Sigma-Aldrich, USA), a D2 receptor agonist, induced a similar activation of Kv7/M-like currents as dopamine did (Figure 3Aiii, *p* = 0.0022, paired *t*-test). Next, we confirmed that this D2-dependent action on Kv7.4 was mediated by G_i/o_ protein since it was blocked by 6–9 h pre-treatment with 500 ng/ml Pertussis toxin (PTX, Alomone Labs, Jerusalem, Israel; Figure 3D, *p* = 0.8652, paired *t*-test). Finally, a redox-dependent step was involved, since DA activation of Kv7.4 was prevented by a reducing agent dithiothreitol (DTT, Ruitaibo, 1 mM; Figure 3E, *p* = 0.1502, paired *t*-test). Thus, the mechanism of DA-induced Kv7.4 channel augmentation and its subsequent inhibitory action appears to be mediated by the oxidative modification of the redox-sensitive Kv7.4 channels (Gamper et al., 2006) in a way similar to that described for the PTX-sensitive augmentation of Kv7 channels by substance P in sensory neurons (Linley et al., 2012).

### An Altered D2-Kv7.4 Signaling Pathway in Social Defeat Model Mice

**Figure 4:**
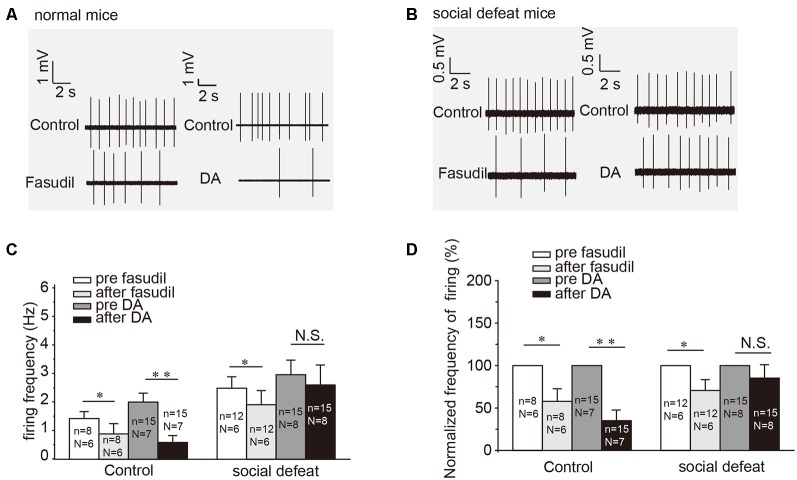
DA-induced inhibition on the firing of NAc-projecting VTA DA neurons is reduced in the social defeat model of mice. **(A,B)** Example traces from cell-attached recordings of NAc-projecting VTA DA neurons are shown before and after administration of fasudil (a selective Kv7.4 opener; 10 μM) and DA (20 μM) in the control and the social defeat model mice. **(C)** Summarized the effect of fasudil and DA on the spontaneous firing frequency of NAc-projecting VTA DA neurons. **p < 0.01, *p < 0.05; N.S., not significant, paired *t*-test. *N* = number of animals; *n* = number of recordings. **(D)** Normalized firing frequency based on the data shown in C. Error bars represent SEM. **p < 0.01, *p < 0.05; N.S., not significant, one-sample Wilcoxon signed ranks test.

Our previous study had established that specific activator of Kv7.4 in the VTA DA neurons was able to ameliorate the increased excitability of VTA DA neurons and the social defeat stress-induced depression-like behavior (Li et al., 2017). In this study, we further tested the involvement of the DA (D2)-Kv7.4 signaling pathway in the development of depression. Indeed, fasudil (the National Institute for the Control of Pharmaceutical and Biological Products, Li et al., 2017), reduced the firing rates of NAc-projecting VTA DA neurons in social defeat model mice by 31.6 ± 12.2% from a basal value of 2.53 ± 0.39 Hz (Figure 4C, *p* = 0.0150, paired *t*-test; Figure 4D, *p* = 0.0156, one sample Wilcoxon signed Ranks test). The reduction of the firing rate was statistically significant, although somewhat less prominent than that in the normal control mice (Figure 4A) by 42.2 ± 13.5% reduction from a basal value of 1.46 ± 0.25 Hz (Figure 4C, *p* = 0.0170, paired *t*-test; Figure 4D, *p* = 0.0313, one sample Wilcoxon signed Ranks test). The validation of social defeat experiments shows in Supplementary Figure S2.

Interestingly, DA failed to affect the firing rates of VTA DA neurons from the social defeat model mice (Figures 4B–D). Given the facts that: (i) fasudil is capable of restoring the VTA DA neuron firing rate (Figure 4C); (ii) fasudil alleviates the depression-like behavior in socially-defeated mice (Li et al., 2017); and (iii) both these effects are Kv7.4-dependent (Li et al., 2017).

## Discussion

The major findings of this present study include the following: (i) Kv7.4 channels strongly contribute to the DA-mediated auto-inhibition of VTA DA neurons in specific-projection pathways; (ii) DA activates Kv7.4 through a G_i/o_-protein-mediated, redox-sensitive signaling cascade; and (iii) reduced DA-mediated auto-inhibition may be involved in the development of social defeat stress-induced depression-like behavior.

DA-mediated auto-inhibition is believed to be induced by a D2 receptor-mediated activation of Kir3/GIRK channels (Beckstead et al., 2004; Courtney et al., 2012). However, it has been shown (McCall et al., 2017) and firmed by us in this study that deletion of Kir3.2 only marginally reduced this effect, suggesting that mechanisms other than Kir3/GIRK must also be involved. Driven by identification of Kv7.4 as another prominent regulatory K^+^ channel in VTA, we tested the possible role of Kv7.4 as an alternative or complementary mechanism of DA auto-inhibition in VTA. Additional arguments to focus Kv7.4 are as follows: (i) D2 receptor activation was shown to potentiate Kv7 channel activity in heterologous expression systems. This effect was most prominent with the Kv7.4, amongst other Kv7 subunit tested (Ljungstrom et al., 2003). (ii) Substance P acting *via* NK1 receptors potentiated Kv7.4 activity in a G_i/o_-mediated manner (Linley et al., 2012), a mechanism utilized in DA activation of Kir3.2. (iii) A direct activation of Kv7.4 by the binding of G_βγ_ dimmer has been reported (Stott et al., 2015).

We found that Kv7.4 clearly contributed to DA-induced inhibition of spontaneous firing of VTA DA neurons. Interestingly, genetic deletion of either Kv7.4 or Kir3.2 in mice did not significantly affect the DA-induced inhibition on VTA DA firing but was significantly reduced in double-KO mice. In addition, DA inhibition was partially recovered by either the Kv7 specific blocker XE991 or by the Kir3 channel blocker, tertiapine-Q (data not shown). Importantly, the recovery of DA-induced inhibition by XE991 (but not tertiapine-Q) was lost in Kv7.4^−/−^ mice and, similarly, the recovery by tertiapine-Q (but not by XE991) was lost in Kir3.2^−/−^ mice. These data strongly indicate that: (i) both channels significantly contribute to the DA-induced inhibition of VTA DA neuron firing. (ii) Based on the pharmacology, Kv7.4-mediated mechanism is perhaps the dominant (yet, this will have to be confirmed with an independent method). (iii) Global genetic deletion of either of the channel is likely to result in compensatory enhancement of the contribution of the remaining channel into the DA inhibition, hence the inhibition is preserved in mice with individual knock-out of either of the channels. However, the genetic deletion of both channels results in only very weak, residual inhibition. It is not clear from this current study which conductance contributes to the remaining DA-induced inhibition. We speculate that other G_i_-coupled protein of ion channel may be involved in the DA auto-inhibition in double KO mice; a recent study reported that activation of D2 receptors strongly inhibited NALCN-mediated sodium leak currents in a G_i/o_ protein-dependent manner, which drive spontaneous firing of DAergic neurons in VTA (Philippart and Khaliq, 2018). Clearly more studies needed to clarify this interesting issue.

We also found the signaling cascade linking DA with Kv7.4 activation, which required G_i/o_ (as it was sensitive to PTX; Figure 3D) and an oxidative step (as it was prevented by a reducing agent, DTT; Figure 3E). Kv7 channels are potentiated by oxidative modification of the triple-cysteine pocket in the channel intracellular S2-S3 linker (Ooi et al., 2013). This mechanism has been suggested to mediate NK1 receptor-mediated potentiation of M channel activity in sensory neurons (Linley et al., 2012). The NK1 pathway was also sensitive to PTX and DTT and targeted of M channels (Linley et al., 2012). Yet, this signaling cascade requires further investigation and other mechanisms could also be at play.

D2 auto-receptor pathways show plasticity and are dynamically regulated by reward circuits and drugs of abuse (Beckstead et al., 2007; Courtney et al., 2012). Here, we found that the VTA DA neurons from the socially defeated mice lost their inhibitory responses to DA, which is consistent with other results reported that social defeat stress reduces the degree of DA-mediated self-inhibition of VTA DA neurons, and also the amplitude of DA-induced outward currents in the VTA (Lo Iacono et al., 2018). We speculate that the coupling between the D2 receptor and Kv7 was altered, or that the D2 receptor was downregulated or desensitized, or mechanisms targeted by microglia after social defeat stress (Ford, 2014; Lo Iacono et al., 2018). One of the interesting experiments planned to be performed in the future study would be to compare the GABA_B_ receptor effect, since the later also inhibit DA neuron firing with a similar mechanism as D2 does. Importantly, since functional Kv7.4 channels are still present in VTA DA neurons (even though at a reduced level), targeting the remaining functional Kv7.4 would bypass this malfunction (Li et al., 2017). Selective targeting of Kv7.4 must, therefore, be considered as a promising antidepressant treatment strategy.

## Data Availability Statement

The datasets generated for this study are available on request to the corresponding author.

## Ethics Statement

All experiments were conducted in accordance with the guides of the Animal Care and Use Committee at Hebei Medical University.

## Author Contributions

HZ conceived, designed and supervised the experiments. MS and LL performed the experiments, acquired and analyzed the data and prepared the figures. JW performed immunofluorescence of brain slice. HS, LZ and CZ performed behavior test. YX, HZ, NG and XD prepared the final version of the manuscript.

## Conflict of Interest

The authors declare that the research was conducted in the absence of any commercial or financial relationships that could be construed as a potential conflict of interest.

## Abbreviations

DA: dopamine
VTA: ventral tegmental area
D2: dopamine type 2
NAc: nucleus accumbens
mPFC: medial prefrontal cortex
BLA: basolateral amygdale
aCSF: artificial cerebrospinal fluid
DAPI: 4,6-diamidino-2-phenylindole
PFA: paraformaldehyde
TH: tyrosine hydrolyze
PTX: perturssis toxin
DAT: dopamine transporter
GIRK: G protein gated inwardly rectifying K^+^ channels.
