# Supplementary material for: Kv7.4 Channel Contribute to Projection-Specific Auto-Inhibition of Dopamine Neurons in the Ventral Tegmental Area
Source: Front Cell Neurosci. 2019 Dec 18;13:557. doi: 10.3389/fncel.2019.00557 (PMC6930245; doi:10.3389/fncel.2019.00557)
Supplement: Supplementary file 1 [file Data_Sheet_1.PDF]

Figure S1

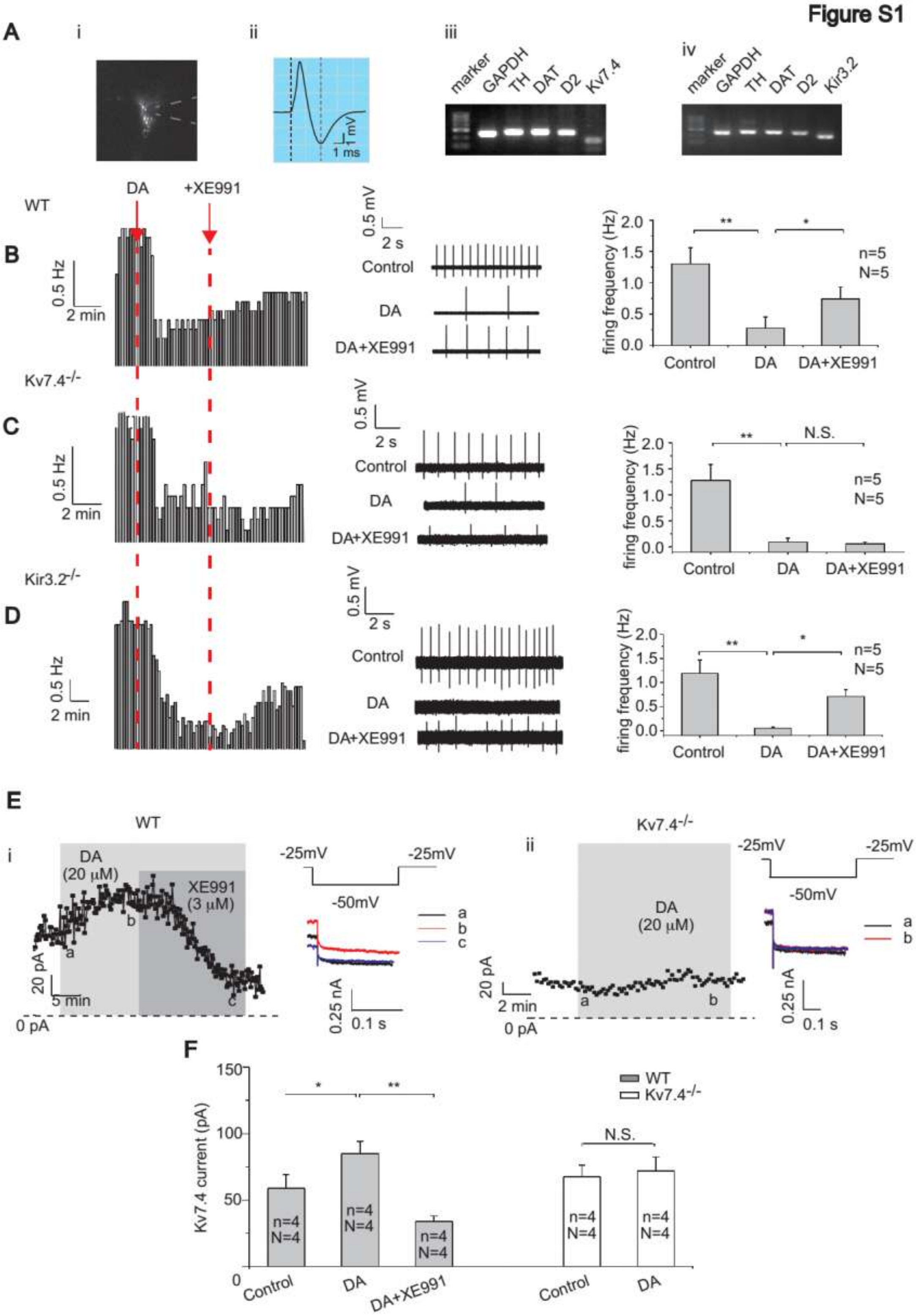

A
i
ii
1 mV
1 ms
iii
marker
GAPDH
TH
DAT
D2
Kv7.4
iv
marker
GAPDH
TH
DAT
D2
Kir3.2
WT
DA
+XE991
B
0.5 Hz
2 min
0.5 mV
2 s
Control
DA
DA+XE991
firing frequency (Hz)
2.0
1.5
1.0
0.5
0.0
**
*
n=5
N=5
Control
DA
DA+XE991
Kv7.4-/-
C
0.5 Hz
2 min
0.5 mV
2 s
Control
DA
DA+XE991
firing frequency (Hz)
**
N.S.
n=5
N=5
Kir3.2-/-
D
0.5 Hz
2 min
0.5 mV
2 s
Control
DA
DA+XE991
firing frequency (Hz)
**
*
n=5
N=5
E
WT
i
DA
(20 μM)
XE991
(3 μM)
a
b
c
20 pA
5 min
0 pA
-25mV
-25mV
-50mV
a
b
c
0.25 nA
0.1 s
Kv7.4-/-
ii
DA
(20 μM)
20 pA
2 min
a
b
0 pA
-25mV
-25mV
-50mV
a
b
0.25 nA
0.1 s
F
Kv7.4 current (pA)
150
100
50
0
WT
Kv7.4-/-
*
**
N.S.
n=4
N=4
Control
DA
DA+XE991
Control
DA

Figure S1. DA activates Kv7/M and Kv7.4 contributes to the DA-induced inhibition of firing in BLA-projecting DA neuron.

(A) Identification of VTA DA neurons projecting to BLA. (i), a single fluorescent neuron with recording pipette (dashed line); (ii), an example action potential waveform of the DA neuron; (iii&iv), single-cell PCR results. **(B-D)** VTA DA neuron firing recorded with loose cell-attached patch recordings. XE991 reversed the DA-induced inhibition of neuron firing in WT (B) and Kir3.2$^{-/-}$ (D) mice, but not in Kv7.4$^{-/-}$ mice (C). Summarized data are presented in **(C). (E and F)** Kv7/M currents recorded from DA neurons of WT (i) and Kv7.4$^{-/-}$ (ii) mice. The time-course of M current amplitudes was shown in (i) and (ii)..Error bars represent SEM. ~~***$p < 0.001$,~~ **$p < 0.01$, *$p < 0.05$, N.S., not significant. One-way repeated measures ANOVA with Bonferroni post-hoc test. N = number of animals; n = number of recordings.
