# Supplementary material for: Kv7.4 Channel Contribute to Projection-Specific Auto-Inhibition of Dopamine Neurons in the Ventral Tegmental Area
Source: Front Cell Neurosci. 2019 Dec 18;13:557. doi: 10.3389/fncel.2019.00557 (PMC6930245; doi:10.3389/fncel.2019.00557)
Supplement: Supplementary file 2 [file Data_Sheet_2.PDF]

Figure S2

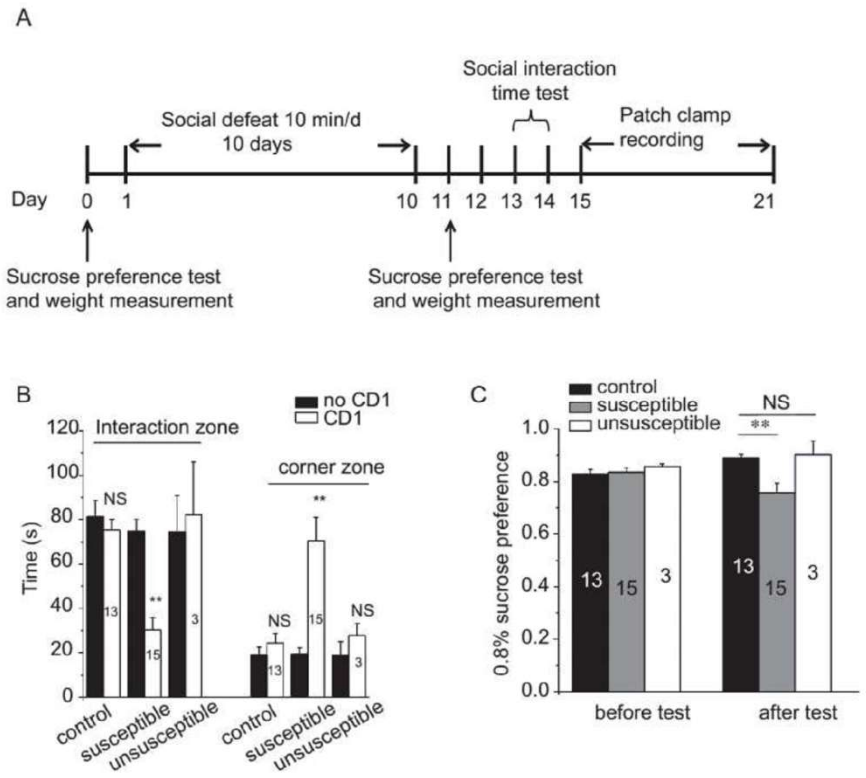

Figure S2. Social defeat mice model of depression.

(A) Timeline of chronic social defeat stress stimulation, behavior tests and electrophysiological recordings. **(B)** The effect of social defeat stress on the length of time mice stayed in the interaction zone and corner zone. For the definition of susceptible and unsusceptible mice, see Method for detail. $^{**}p < 0.01$, paired t-test N.S. not significant, compaired with 'no CD1'. **(C)** The effect of social defeat stress on the preference rate of sucrose ($^{**}p < 0.01$, one-way ANOVA. N.S. not significant, compaired with control mice). Numbers in the column indicate number of animals.
